# Effects of Acidic Challenge on Demineralized Root Surface Treated with Silver Diamine Fluoride and Potassium Iodide

**DOI:** 10.3390/diagnostics13030530

**Published:** 2023-02-01

**Authors:** Alexandru Iovan, Marcelin Benchea, Simona Stoleriu, Ionuț Tărăboanță, Nicanor Cimpoeșu, Irina Nica, Sorin Andrian

**Affiliations:** 1 Faculty of Dental Medicine, “Grigore T. Popa” University of Medicine and Pharmacy, 16 Universitatii Str., 700115 Iași, Romania; 2 Faculty of Mechanical Engineering, “Gh. Asachi” Technical University, 67 Dimitrie Mangeron Str., 700050 Iași, Romania; 3 Faculty of Materials Science and Engineering, “Gh. Asachi” Technical University, 67 Dimitrie Mangeron Str., 700050 Iași, Romania

**Keywords:** silver diamine fluoride SDF, hardness, root caries, remineralization, SEM, EDX

## Abstract

Background: The aim of the study was to assess the protective effect of applying potassium iodide (KI) over silver diamine fluoride (SDF) on demineralized root dentin in the case of a sustained acidogenic attack. Methods: Forty caries-free third molars were used in the study. A diamond disc was used to separate the roots and the tooth crowns from the roots. Each root fragment was randomly distributed in one of the four study groups: C—samples were not demineralized; DD—demineralized samples; RS1—demineralized samples covered with SDF+KI (RS-Riva Star product, SDI limited, Bayswater, Australia); RS2—demineralized samples covered with SDF+KI and submersed to another acidic challenge for 3 days. SEM and EDX were used for the morphological and elemental analysis. Vickers hardness assessment was performed using a tribometer CETR UMT-2 (Bruker Corporation, Berlin, Germany). One-way ANOVA and post hoc Bonferroni tests were used for the statistical analysis with a significance level of *p* < 0.05. Results: Morphological and elemental changes were observed on the surface of the study samples. Significant differences were observed between the recorded hardness values of groups C and DD (*p* = 0.005), C and RS2 (*p* = 0.002), DD and RS1 (*p* = 0.011); RS1 and RS2 (*p* = 0.004). Conclusions: The application of SDF and KI (Riva Star product) on root dentin caries resulted in the formation of a heterogeneous outer layer that sealed the dentin and increased the microhardness of the treated surface. In the conditions of the present study, this layer did not provide enough protection for root dentin exposed to continuous attacks.

## 1. Introduction

Root caries is a pathology inevitably associated with periodontal disease [1]. With the increase in life expectancy and the advances in dentistry, the number of teeth with periodontal recessions and susceptibility to root caries has significantly increased [2,3]. The treatment of carious root surfaces can be more difficult compared to coronal surfaces due to the complexity of the local context (cementum/root dentin/marginal periodontium), as well as the debilitating pathology associated with patients’ age.

For this reason, the therapeutic management of root caries is oriented towards minimally invasive strategies and the promotion of arresting root lesions by controlling etiological factors [4]. Non-restorative caries control is a recently proposed treatment alternative for root caries [5]. This strategy aims at exposing the lesion to cleaning and self-cleaning agents and applying varnishes that reduce carious activity and promote remineralization [6]. The efficiency of the method in controlling the dental caries of primary teeth has been evaluated by several studies [7,8], but the scientific evidence for the root caries treatment is relatively scarce. Treatment recommendations in active root lesions suggest the application of highly concentrated fluoride varnishes (>20,000 ppm) and silver diamino fluoride (SDF > 30%), although these recommendations are based on inconclusive evidence [4].

The presence of active root caries is associated with the existence of a highly cariogenic oral environment. Elderly patients and those with debilitating systemic diseases, who represent the main risk group, show a reduced capacity for radical changes in dietary and oral hygiene habits [9]. The success of non-restorative therapy involves changing the oral environment conditions in favor of remineralization phenomena. In the absence of this change, it seems unlikely that non-operative treatment will be sufficient to stop the lesions. The application of SDF is one of the proposed alternatives for preventing and arresting root caries [9,10]. Combining SDF with potassium iodide (KI) was proposed to prevent SDF-induced post-therapeutic staining [11,12], but the long-term effectiveness of this combination has not been validated by sufficient clinical and laboratory studies. This study aimed to analyze whether such a material protects the demineralized root dentin in the case of a sustained acidogenic attack, characterized by prolonged periods in which the pH drops below the critical value.

Therefore, the effects of treatment with SDF and KI root dentin hardness, chemical composition, and morphology of the demineralized root surface were evaluated immediately after the application of the material and after exposure to a prolonged cariogenic attack. The null hypothesis was that SDF+KI application and the subsequent exposure to cariogenic attack do not influence the physical and chemical properties of root dentin.

## 2. Materials and Methods

### 2.1. Sample Preparation

G*Power software (version 3.1.9.7., Heinrich Heine-Universität Düsseldorf, Düsseldorf, Germany) was used for the sample size calculation. An effect size of 0.25 was used, which is a medium effect according to Cohen’s classification. The alpha value was 0.05 with a power of 95%. The results estimated a total number of 79 required samples.

**Figure 1:**
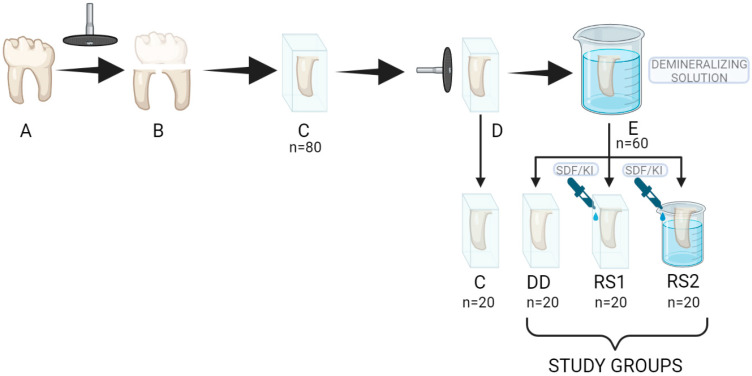
Sample preparation steps. (**A**) caries-free third molar; (**B**) separation of crown and roots with a diamond disc; (**C**) root fragments embedded in blocks of self-curing acrylic resin; (**D**) root surfaces planed and polished with extra-thin abrasive discs; (**E**) samples in groups (**DD**,**RS1**,**RS2**) stored in a 10 mL demineralizing solution.

For the present study, forty caries-free third molars were used, extracted in accordance with a protocol (no. 89/07 June 2021) approved by the Research Ethics Committee of “Gr. T. Popa”, University of Medicine and Pharmacy of Iasi, Romania. Collected teeth were cleaned by manual scaling and brushing with polishing paste Universal Polishing Paste (Ivoclar Vivadent, Schaan, Lichtenstein) and then stored in a 0.5% chloramine T solution at 4 °C until testing. A diamond disc (DFS-DIAMON GmbH, Riedenburg, Germany) activated at 5000 rpm under running water was used to separate the roots and the tooth crowns from the roots (Figure 1B). Each root fragment was distributed to one of the 4 study groups described in Table 1.

The root fragments were then embedded in blocks of self-curing acrylic resin (Duracryl Plus, Spofa Dental, Jičín, Czech Republic) so that only one root surface was exposed (Figure 1C). The surfaces were then planed and polished with Sof-Lex^TM^ Extra-thin abrasive discs (3M ESPE Dental Products, St. Paul, MN, USA) with decreasing grit (Figure 1D). The samples were cleaned of detritus in an ultrasonic bath for 5 min. A layer of acid-resistant nail varnish was applied to the prepared surfaces in order to obtain a 4 mm × 3 mm area of exposed root dentin.

Samples in groups DD, RS1, and RS2 were stored in a 10 mL demineralizing solution (Figure 1E) containing 0.2 M lactic acid, 3.0 mM CaCl_2,_ and 1.8 mM KH_2_PO_4_ with a pH of 4.5 for 3 days at 37 °C to simulate artificial root caries lesions [13,14]. The solution was changed each 24 h in order to keep a constant pH. The pH was verified every 12 h with a pH meter (Thermo Scientific Eutech pH 5+, Vernon Hills, IL, USA).

After the demineralization process, the samples were rinsed thoroughly with distilled water and gently air-dried without desiccating the dentin. Then, each sample in groups DD, RS1, and RS2 was subjected to one of the 3 experimental protocols on the exposed root surface (Table 1), after which they were stored for 24 h in distilled water until evaluation by scanning electron microscopy (SEM) and spectroscopy (EDX) or hardness evaluation.

The composition and the application protocol of the Riva Star product are presented in Table 2 and Table 3.

### 2.2. SEM Evaluation

Three samples in each group were morphologically evaluated under a Scanning Electron Microscope (Vega Tescan LMH II, Tescan, Brno, Czech Republic) with operating conditions of 10–30 kV, 15.5 WD. Secondary electrons (SE) detector was used to obtain the images. There were no phase differences in the material, and the variation in the chatode power permitted the correct biological material evaluation.

### 2.3. EDX Evaluation

Elemental analysis was realized on three samples in each study group by X-ray dispersive spectroscopy using an EDX X Flash 6L10 detector, Esprit 2.2 (Esprit, Bruker Corporation, Berlin, Germany) soft wave in automatic mode/list of elements, and Line, Point and Mapping sought to detect the presence and distribution of calcium, phosphorus, silver, potassium, iodine, and fluorine. Energy-dispersive spectroscopy experiments (Automatic-Precise mode, PB-ZAF detection type with 0.01% detection limit) were realized with repetition on different areas in order to obtain average values (five times). Esprit 2.2 software was used to evaluate the energies registered. Standard deviation of the elements’ percentages was established (from five determinations of chemical composition percentages on the same area 0.25 mm^2^) for each chemical element. Absolute and relative errors of the detector were presented in order to compare the results.

### 2.4. Microhardness Test

The surface microhardness of 10 samples in each group was assessed using a tribometer CETR UMT-2 (Bruker Corporation, Berlin, Germany). A Vickers-type indenter was used for the microindentation test (diamond cone with an angle of 120° and a tip with a radius of 200 μm). The samples were fixed on the flat plate of the tribometer, and the indentations were made by pressing the indenter with a vertical force of 5 N with an indentation speed of 0.005 mm/s, a preload time of 15 s, a charging time of 30 s, a holding time of 15 s, and a download time of 30 s. The software (Tribometer CETR UMT-2, Version 1.01 software, Bruker Corporation, Berlin, Germany) performed the automatic test and recorded the vertical load Fz, the time, and the vertical displacement distance C of the indenter (depth of penetration). Hardness was automatically calculated from the discharge slope curve and expressed in GPa.

### 2.5. Statistical Analysis

IBM SPSS 26.0.0. software (SPSS Inc., Chicago, IL, USA) was used for statistical analysis. Levene’s test was used to assess the homogeneity of variances, and then statistical analysis was performed using one-way analysis of variance (ANOVA) and post hoc Bonferroni tests with a significance level of *p* < 0.05.

## 3. Results

### 3.1. SEM Evaluation Results

**Figure 2:**
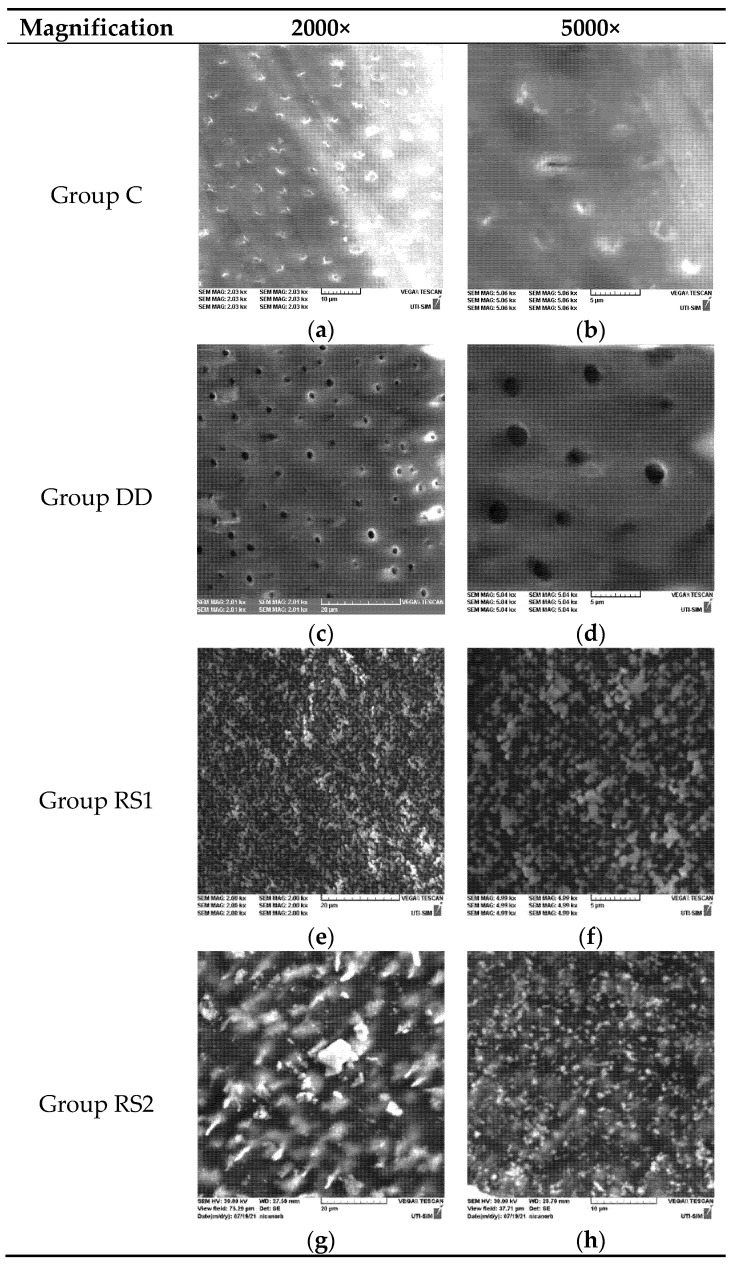
SEM images of a sample in each group (C, DD, RS1, RS2) at 2000× and 5000× magnifications.

The morphological analysis of dentin subjected to demineralization (group DD) showed the dentin surface with opened tubules and the absence of a smear layer (Figure 2c,d).

The morphological analysis of the samples in group RS1, consisting of demineralized dentin samples treated with RS, revealed deposits with a heterogeneous appearance on the dentin surface. Most images show areas of dentin completely covered with uniform deposits with a granular appearance (Figure 2e,f). On all samples, including areas where inconsistent deposits were observed, the dentinal tubules appeared completely occluded with a precipitate or crystalline-looking particles.

For the samples in group RS2, the images showed less consistent material deposition. The deposits on the surface were uneven. The particles had variable sizes and were more dispersed compared to the images of the samples treated with RS immediately after application (Figure 2g,h).

### 3.2. EDX Evaluation Results

For the samples in group C, EDX analysis showed the presence of calcium and phosphorus on the samples’ surface. The analysis confirmed high mass concentration and normalized weight percent of oxygen, followed by carbon and calcium, and lower concentrations of phosphorous. An example of EDX elemental analysis on the surface of one sample in group C is presented in Table 4.

EDX analysis of the samples in group DD also showed high mass concentration and normalized weight percentage of carbon and oxygen, followed by nitrogen. Calcium and phosphorus were detected in lower concentrations than in group C. An example of EDX elemental analysis on the surface of one sample in group DD is presented in Table 5.

For the samples in group RS 1, EDX analysis showed the presence of high concentrations of iodine, followed by silver, and lower concentrations of potassium on the samples’ surfaces. In contrast, fluorine was almost undetectable (mass concentration and normalized weight percent were lower than the values of absolute and relative errors of detection), and calcium and phosphorus were undetectable. An example of EDX elemental analysis on the surface of one sample in group RS1 is presented in Table 6.

For the samples in group RS2, EDX analysis confirmed the presence of iodine and silver on the surfaces of samples with higher concentrations of iodine, followed by silver, while potassium and fluorine were undetectable. Calcium and phosphorus were observed in amounts close to the detection limit. An example of EDX elemental analysis on the surface of one sample in group RS2 is presented in Table 7.

### 3.3. Microhardness Test Results

**Figure 3:**
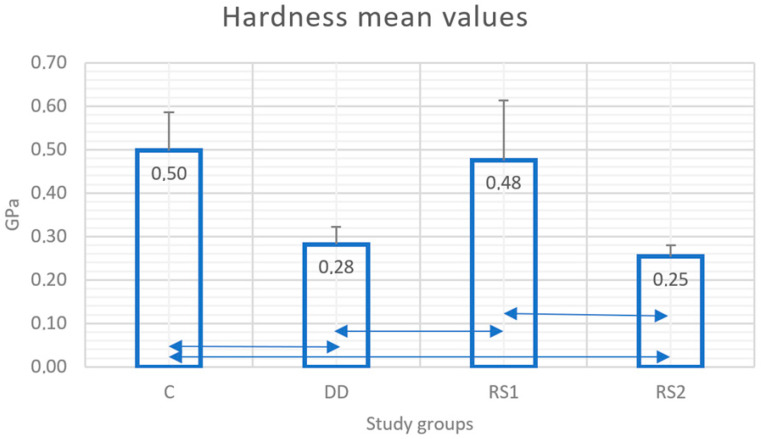
Mean values (Gpa) and standard deviations (SD) of hardness in each study group. Double arrow lines (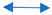) show statistical differences between groups.

Hardness mean values and standard deviations for all the groups are presented in Figure 3. It can be observed that control group C reached the peak with a value of 0.50 GPa, followed by groups RS1 with 0.48 GPa, DD with 0.28 GPa, and RS2 with 0.25 GPa. Statistically significant differences were recorded between groups C and DD (*p* = 0.005); C and RS2 (*p* = 0.002); DD and RS1 (*p* = 0.011); RS1 and RS2 (*p* = 0.004).

## 4. Discussion

Ample evidence supports the efficacy of high-concentration fluoride in the management of root caries [15]. In the presence of an organic dentinal matrix, the mechanism of remineralization is based on the absorption of remineralizing ions into the underlying demineralized tissue rather than precipitation on the surface [16]. When subjected to immediate acid or mechanical attack, these materials can significantly diminish their effectiveness. Therefore, this study aims to evaluate tooth behavior in a demineralizing environment that simulates an intense and prolonged cariogenic attack immediately after the application of the remineralizing material. To simulate artificial root caries lesion and acidic attack after remineralization, we used a demineralization solution that had been also applied in other previous studies [12,17].

SDF promotes remineralization and increases the resistance of dental tissues to acid attack by forming calcium fluoride and hydroxyapatite deposits [18], inhibits microbial activity mainly due to silver ions [19], and prevents the degradation of dentinal collagen by inhibiting the activity of collagenases and cysteine-cathepsins [20]. The material tested in this study uses a combination of SDF with KI. Potassium iodide was introduced more recently to prevent the staining of dentin caused by unreacted silver ions after exposure to light.

In our study, SEM images and EDX analyses revealed the presence of a heterogeneous layer with high concentrations of silver and iodine that covered demineralized dentin treated with RS immediately after application. The dentinal tubules were consistently occluded by granular deposits, regardless of the area examined. This aspect is consistent with the results of a previous study that showed the formation of dense, granular, clustered structures after SDF treatment [21]. The EDX analysis did not provide sufficient data to determine the chemical composition of the layer, but it showed the presence of iodine, silver and potassium on the surface in proportions that varied depending on the morphology of the examined areas.

The surface morphology was modified after acid attack, with increased dispersion of the deposits on the dentin surface. Elemental EDX analysis confirmed the decrease in silver and iodine concentrations on the surface of RS-treated samples after the acid attack. Previous EDX studies suggest that the metal crystals precipitated on the surface of the specimen may be AgCl salts [15] with irregular shapes [22], but in our study, the areas with granular agglomerations showed high concentrations of silver and iodine while chlorine proportions were below the detection limit. The explanation is probably related to the storage medium that had been used. In our study, EDX analysis revealed only high concentrations of silver and iodine in the crystalline particles, probably as a result of the RS mechanism combining SDF and KI during application, leading to AgI formation and reducing the silver ions available for reaction with dentin [23,24]. The low phosphorus concentrations found in this study suggest that if silver phosphate was formed during the reaction with hydroxyapatite, it was probably present in deeper subsurface areas. Another explanation could be related to the sensitivity of the detection method and especially to the time required for the silver phosphate crystals to become mature [25].

An unexpected result was the low amount of fluoride detected after RS application. Traces of fluoride were almost undetectable in the outer layers of both experimental groups. Normally, a 38% SDF solution contains 44,800 ppm of fluoride [26]; therefore, calcium fluoride-like compounds are expected to be formed after application [27]. However, a previous in vitro study also found that silver and fluoride were inconspicuous in active carious lesions treated with SDF [28], and other laboratory studies showed decreased fluoride content after water washing [29] or could not find calcium fluoride on demineralized dentin treated with SDF after pH-cycling [22,30].

The lack of fluorine detection can be explained by a rapid washing effect caused by the high fluidity of SDF solutions [29,31] or by the high detection limit of EDX [32]. Moreover, the caries model with a continuous acid challenge without de-/remineralization cycles and the short storage period could contribute to the accelerated dissolution of calcium fluoride without the formation of fluorapatite crystals.

To validate the SEM and EDX analysis results, mechanical tests were performed. Rehabilitation of mechanical properties is considered a good indicator of functional remineralization of damaged dentin [33,34,35].

In our study, the application of RS produced a significant increase in the hardness values of demineralized dentin immediately after application. However, the treatment was not able to increase the microhardness to values similar to those of sound dentin, a conclusion that has been confirmed by a previous study performed on demineralized bovine dentin [36]. Other previous studies confirmed the improvement in the mechanical properties of the demineralized dentin after SDF+KI treatment [15,37]. Knoop hardness, modulus of elasticity, and creep behavior improved after the application of SDF+KI on the dentin demineralized by storage for five days in a similar solution [15]. Other studies also concluded that SDF improved the properties of artificial demineralized dentin even when compared to other fluoride varnishes [37,38].

In the present study, the remineralization effect was not fully maintained after subjecting the samples to a demineralization challenge. The surface microhardness significantly decreased after the storage of the samples treated with RS in acid. In other in vitro studies performed on dentin affected by caries, SDF treatment caused a significant increase in the immediate dentin hardness, which the authors correlated with an increased surface mineralization [21,39,40]. Contrary to our results, this effect persisted after repeated acid attacks simulated by pH cycling for 8 days in de- and re-mineralization solutions [21,41]. These conflicting results can be explained by the pH cycling model allowing some remineralization to occur. In contrast, the continuous and sustained demineralizing attack simulated in our study did not allow these reactions to take place, with the protecting layer being corroded continuously by the acidic solution.

The results of the mechanical tests confirmed the images observed in the scanning electron microscope and EDX analysis, which showed a deterioration of material deposited on the demineralized dentin surface after storage in the demineralizing solution. However, the microhardness values did not significantly decrease below the initial values of demineralized dentin (before the treatment with RS), supporting the hypothesis that RS had a moderate protective effect on root dentin by slowing down its deterioration when acidic challenge persists.

The limitations of our study are primarily related to the chemical model used for caries simulation and the detection limits of SEM and EDX analysis. Within these limits, our results support the effectiveness of SDF+KI in protecting demineralized root dentin against cariogenic attack immediately after application and the drastic reduction in this effect if pH conditions are maintained below the critical level after application of the material. Further studies are needed to evaluate the effectiveness of SDF+KI application on root dentin remineralization in caries models using pH cycling and longer observation periods.

Even with the limitations of the chemical caries model that we used, the results support the hypothesis that this protection is limited under continuous cariogenic or erosive environmental conditions. It seems likely that in the absence of oral allostasis, when the control of risk factors is inefficient, only the application of such materials to root carious surfaces would not stop the progression of root carious lesions. Further studies are needed to evaluate the effect of repeated application of these materials on carious root dentin under simulated conditions of a strongly cariogenic or erosive environment.

## 5. Conclusions

The application of silver diamine fluoride and potassium iodide on root dentin caries resulted in the formation of an heterogeneous outer layer that sealed the dentin and increased the microhardness of the treated surface.

In the conditions of the present study, this protective layer did not provide enough protection for root dentin exposed to continuous attacks.

## Figures and Tables

**Table 1:** Distribution of the samples in groups.

Study Group	Study Protocol
C (*n* = 20)	Control group: Samples were submersed in distilled water
DD (*n* = 20)	Samples were submersed in demineralizing solution for 3 days
RS 1 (*n* = 20)	Samples were submersed in demineralizing solution for 3 days Riva Star product (RS) (SDI limited, Bayswater, Australia) was then applied on the exposed dentin surface
RS 2 (*n* = 20)	Samples were submersed in demineralizing solution for 3 days Riva Star product (RS) (SDI limited, Bayswater, Australia) was then applied on the exposed dentin surface Samples were stored in distilled water for 24 h Samples were submersed in demineralizing solution for 3 days

**Table 2:** Composition of the Riva Star product.

Product	Composition	Batch No.
Riva Star Capsule Kit (SDI Limited Australia)	Riva Star Step 1: 38% silver diaminofluoride (SDF) Riva Star Step 2: potassium iodide (KI)	1164696

**Table 3:** Application protocol of Riva Star product.

Protocol
Riva Star Solution Step 1 was applied on the exposed dentin surface Riva Star Solution Step 2 was immediately applied to the treated surface until the creamy-white precipitate became clear.

**Table 4:** EDX elemental analysis on the surface of a study sample in group C.

Element	Mass Norm. (%)	Atom (%)	abs. Error (%) (1 Sigma)
Oxygen	46.12745	53.05732	7.906657
Calcium	24.93783	11.45097	0.931029
Carbon	19.5091	29.89149	4.159093
Phosphorus	9.425618	5.600225	0.495739
Sum	100	100	

**Table 5:** EDX elemental analysis on the surface of a study sample in group DD.

Element	Mass Norm. (%)	Atom (%)	abs. Error (%) (1 Sigma)
Carbon	46.2307	54.4655	6.85517
Oxygen	34.19054	30.23935	5.822515
Nitrogen	12.40779	12.53513	3.712765
Calcium	4.972625	1.755698	0.185945
Phosphorus	2.198341	1.004318	0.123339
Sum	100	100	

**Table 6:** EDX elemental analysis on the surface of a study sample in group RS1.

Element	Mass Norm. (%)	Atom (%)	abs. Error (%) (1 Sigma)
Iodine	46.34	20.72	3.90
Silver	35.93	18.90	2.30
Oxygen	16.38	58.10	3.70
Potassium	1.13	1.64	0.57
Fluorine	0.22	0.64	0.73
Sum	100	100	

**Table 7:** EDX elemental analysis on the surface of a study sample in group RS2.

Element	Mass Norm. (%)	Atom (%)	abs. Error (%) (1 Sigma)
Oxygen	59.03	90.55	3.012
Iodine	23.05	4.46	0.31
Silver	15.67	3.56	0.23
Calcium	1.96	1.20	0.10
Phosphorus	0.29	0.23	0.08
Sum	100	100	

## Data Availability

Not applicable.
